# Difference in Particle Transport Between Two Coastal Areas in the Baltic Sea Investigated with High-Resolution Trajectory Modeling

**DOI:** 10.1007/s13280-013-0397-3

**Published:** 2013-04-26

**Authors:** Hanna Corell, Kristofer Döös

**Affiliations:** 1 Department of Biology and Environmental Sciences, University of Gothenburg, Box 461, 405 30 Gothenburg, Sweden; 2 Department of Meteorology, Stockholm University, 106 91 Stockholm, Sweden

**Keywords:** Sediment, Particle transport, Particle-tracking model, Resuspension, Lagrangian, Residence time

## Abstract

A particle-tracking model based on high-resolution ocean flow data was used to investigate particle residence times and spatial distribution of settling sediment for two geo-morphologically different Swedish coastal areas. The study was a part of a safety assessment for the location of a future nuclear-waste repository, and information about the particle-transport patterns can contribute to predictions of the fate of a possible leakage. It is also, to our knowledge, the first time particle-transport differences between two coastal areas have been quantified in this manner. In Forsmark, a funnel-shaped bay shielded by a number of islands, the average residence time for clay particles was 5 times longer than in the modeled part of Simpevarp, which is open to the Baltic Sea. In Forsmark, <10 % of the released particles left the domain compared to 60–80 % in Simpevarp. These site-specific differences will increase over time with the differences in land uplift between the areas.

## Introduction

Analyses of the transport of sediment particles have numerous applications in science and environmental management, e.g., how flow in culverts, over dams, and around bridge piers may cause erosion or how sediment deposition in dams and regulated rivers affects the flow. Sediment particles can also act as carriers of pollutants and nutrients, and modeling their deposition and resuspension is, therefore, of great interest. The use of Lagrangian particle tracking for modeling sediment has become increasingly common as the reduced cost of computer time and memory has made it feasible to couple fully dynamic 3D circulation models with a vast number of concurrent particle-trajectory calculations (see, e.g., Warner et al. 2008, 2010). An alternative method is to use an off-line set-up, where output fields from circulation models are stored and used repeatedly (North et al. 2006, 2011; Kling and Döös 2007). Lagrangian methods provide accurate and efficient means of resolving advection-dominated problems by essentially eliminating the effects of numerical dispersion and artificial oscillations sometimes associated with higher order Eulerian methods (Spivakovskaya et al. 2007). Individual-particle tracking permits visualization of settling and exiting patterns and can provide complementary information to that from Eulerian diffusion-based models.

This study is a comparative analysis of two Swedish coastal areas, where an off-line particle-tracking model is added to a circulation model. The purpose of the experiment was to do a site-specific investigation of how the sediment-transport patterns differed between the two morphologically very different areas. At the time of the study both areas were being examined by the Swedish Nuclear Fuel and Waste Management Co. (SKB) with the aim of finding a suitable site for a future underground nuclear repository (Lindborg et al. 2006). The results were to be used as part of the background information that would be the basis of the decision about which area to choose for further investigations.

The residence times for the sediment particles until exiting the domain or until the first settling event were calculated as a measure of the difference between the areas. The concept of residence time has long been used to describe water exchange, and the calculations can be based either on mass or on time. Here we used time-based analysis with trajectories, where particles are released and their passage through defined boundaries is timed (Döös et al. 2004; Jönsson et al. 2004, 2011). This study is, to our knowledge, the first one in which the residence times in a domain have been calculated for particles in this manner. Based on previous investigations of the water residence times using the same method (Engqvist and Andrejev 1999; Engqvist 2006), we expected the more secluded Forsmark domain to show significantly longer residence times and a larger part of the particles to be retained in the area.

## Materials and Methods

### Study Areas

**Fig. 1:**
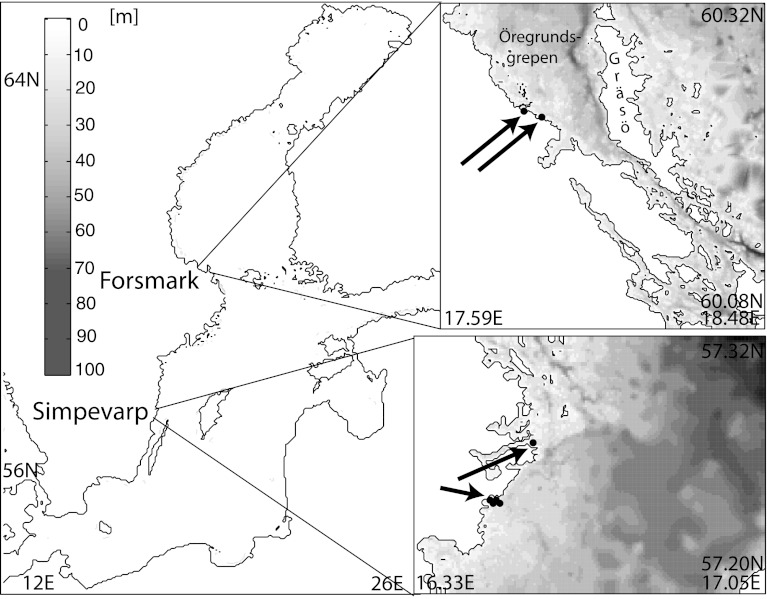
The model bathymetry of the Forsmark (*top right*) and Simpevarp (*bottom right*) coastal regions, and their location in the Baltic Sea area. *Black dots* The starting points for the trajectory simulations

**Fig. 2:**
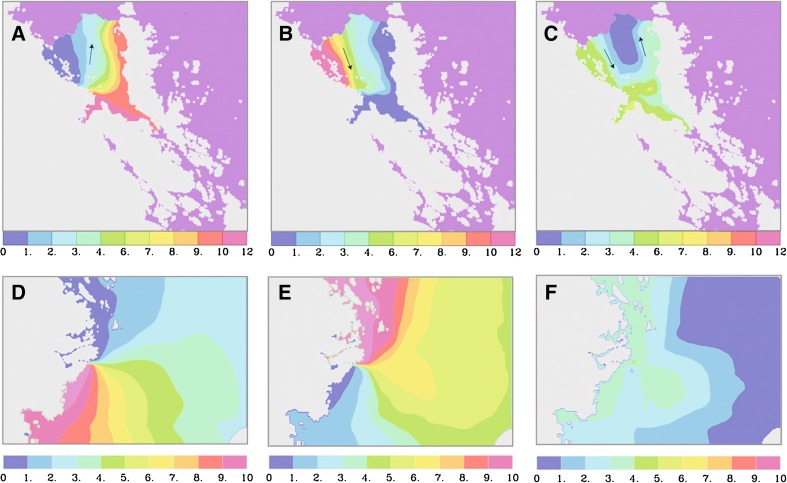
The Lagrangian stream functions for the water exchange in Forsmark (*top*) and Simpevarp (*bottom*). A stream function of this type is determined by following water trajectories, originating from a section along the coast, forwards and backwards in time. Thereafter, the non-divergent volume transport across the grid box walls is calculated; for a technical explanation see, e.g., Appendix A in Jönsson et al. (2011). **a**, **d** Outflow; trajectories are followed from a section along the coast until they leave the bay or the domain, **b**, **e** inflow trajectories calculated backwards in time into the release area, **c**, **f** the combination of outflow and inflow paths. The flow direction is along the streamlines with a constant volume transport between each pair of adjacent streamlines; narrower space between streamlines indicates stronger currents. Values in 100 m^3^ s^−1^

The two areas examined in this study, Simpevarp and Forsmark, are located on the Swedish coast of the Baltic Sea (Fig. 1). The Baltic is a brackish intercontinental sea with essentially no tidal fluctuations (Leppäranta and Myrberg 2009) and the sedimentation processes are thus almost solely controlled by currents and the additional motion at the bottom due to surface waves. Forsmark is dominated by Öregrundsgrepen, a funnel-like embayment with a wider opening toward the north. The narrow southern end is shallow with a threshold depth of approximately 25 m. The retention time for water in Öregrundsgrepen is found to be 12 days for surface waters and about 25 days for bottom water (Engqvist and Andrejev 1999). The thermocline is located at a depth of around 20 m and, the Baltic being a brackish sea, the seasonal thermocline is far more influential for the stratification than the halocline (Leppäranta and Myrberg 2009). The Simpevarp area (Fig. 1) is located slightly northwest of the northern tip of the island Öland. In contrast to Forsmark it is open and has a rapid water exchange with the rest of the Baltic Sea of about 4 days (Engqvist 2006). The water is slightly more saline than that in Forsmark, viz. 7 and 6 psu, respectively (Leppäranta and Myrberg 2009). Most of the eastern part of the domain is deeper than 30 m and in the northeastern part depths of around 100 m are reached. Figure 2 shows the mean flow in the Öregrundsgrepen and Simpevarp domains, represented by Lagrangian stream functions. In Öregrundsgrepen, the flow is strongest in the channel just west of Gräsö island which shields the embayment, although a considerable part of the inflow follows the mainland coast from the north. In Simpevarp, the main flow direction is southwards along the coast. The Simpevarp coastal area also has a number of narrow, more or less land-locked embayments, which are not resolved in the model set-up in this study. See Eriksson and Engqvist (2013) for a quantification of the retention times of the water with these included.

### The Particle-Tracking Framework

The particle-tracking model consists of a trajectory model that calculates the Lagrangian displacement of water parcels based on the output of a 3D ocean circulation model. To this framework a simplified sedimentation and resuspension scheme are attached.

The ocean circulation model that generated the velocity fields used in this study is denoted AS3D (Andrejev and Sokolov 1990). It is a time-dependent, 3D-model with a free surface, based on the Navier–Stokes equations. The present set-up has previously been used for studying the water exchange of Öregrundsgrepen (Engqvist and Andrejev 1999; Engqvist et al. 2006) and the Simpevarp area (Döös and Engqvist 2007) and has a horizontal resolution of 0.1 nautical miles (approximately 185 m) and a vertical resolution ranging from 2.5 m in the surface to 5 m in the deeper parts. The model was forced by gridded downscaled meteorological data of air temperature, pressure, geostrophic wind, and precipitation. At the boundaries it was nested within a coarser (5 nautical miles) Baltic model. This coarser model provides data of density, temperature, and sea-level elevation at the boundaries. The effects of wind stress and bottom friction were included in AS3D in the form of boundary conditions and no-slip conditions are applied at all solid boundaries, except where there is a river discharge (Engqvist and Andrejev 1999). The complete set of equations of the AS3D model including boundary formulation and numerical scheme is given in Andrejev et al. (1997). The model was integrated for one full year for each of the two domains and the output data were stored every hour. The modeled years were selected for being average regarding local temperature and freshwater discharge. AS3D has been tested and validated against measured data from the Baltic Sea (Engqvist and Andrejev 2003) and the model set-up of the areas examined in this study (see Fig. 1) has previously been thoroughly described and validated (Engqvist and Andrejev 1999; Engqvist et al. 2006; Döös and Engqvist 2007).

Hourly data fields of horizontal velocity, density, and temperature from the AS3D model were used as input data to the off-line trajectory model TRACMASS (Döös 1995; Blanke and Raynaud 1997; de Vries and Döös 2001). To determine the trajectory of a given particle the velocities at the sides of each grid box are interpolated to its position, after which the particle path within the box is determined analytically. For a full description of the TRACMASS algorithms see Döös (1995). TRACMASS has the capability of keeping records of all released water particles, thereby making it possible to undertake statistical analyses of, for example, the different ages of particles. The residence time is found by calculating the time difference between the domain entry and exit for each particle and averaging over all particles. Studies using this method for calculating residence times for water have previously been undertaken with TRACMASS for the Baltic Sea (Döös et al. 2004) and the Bay of Gdansk (Jönsson et al. 2004).

### Sedimentation and Resuspension Parameterization

The model settling velocity was calculated using Stokes’ law, *w*_s_ = *gd* ^2^(*ρ*_s_ − *ρ*_w_)/18*μ* (where *ρ*_s_ and *d* are the particle density and diameter, respectively, and *ρ*_w_ and *μ* are the water density and viscosity, and *g* is the acceleration due to gravity, the formulae valid for viscous settling of particles with *d* < 0.2 mm). This “quiescent-water settling velocity” *w*_s_ was added to the vertical component of the AS3D model velocity field at the location of the sediment particle for each time step. The modeled particle sizes, clay and silt, often form larger flocs or aggregates and Stokes’ law is not always applicable for these larger particles. One way around this is to use an empirical formula to model a range of particle diameters and densities (Jiménez and Madsen 2003). However, we chose the simplest possible but still physically consistent approach and used the Stokes-law idealization. This can be interpreted as the suspended-sediment concentration and/or the salinity being low, thus limiting the flocculation. As the Baltic is a brackish sea and the particle-release points did not coincide with any river outlets or other areas where the particle concentration is expected to be high, this approximation was found to be acceptable. The calculated settling velocity varies with the density and viscosity of the water, and turned out to be between 0.05 and 1.0 mm s^−1^. This is within the range of in situ measurements of natural sediments in fresh and brackish water (Mikkelsen and Pejrup 2001; Lumborg 2005; Maa and Kwon 2007).

Resuspension of a settled particle will take place if the shear stress at the bottom exceeds a threshold value. The particle will then be lifted up a short distance above the bottom and be caught in the flow field again. As the shear stress at the bottom is not an output variable of the AS3D circulation model, the horizontal velocities at the lower boundary of the bottom grid box were used as proxies. With almost no tidal influence in the Baltic Sea, and as the fetches within the modeled area that affect the surface waves are short, the largest part of the bottom shear is expected to be induced by the circulation. The threshold velocity for entrainment was taken from Postma (1967), stating a critical velocity of about 10 cm s^−1^ for inducing resuspension for the modeled particle sizes. No sediment layers at the bottom are included in this model; a particle rests directly on the “floor” of the bottom grid box and does not interact with the bottom material. Both erosion and deposition can occur simultaneously and there is no explicit critical limit in the model when deposition occurs; when the particle reaches the sea floor and no longer is subjected is to any horizontal motion, it will settle. The particle can move around just above the bottom as long as there is water motion strong enough to carry it.

### Experiment Set-Up

The model simulations were started from a number of points (cf. Fig. 1), where groundwater carrying radionuclides from such a repository could debouch into the marine coastal zone (Lindgren et al. 2001). Two sets of simulations were made: one with clay particles (diameter 1 μm) and one with silt particles (diameter 10 μm), both with a density of 2620 kg m^−3^ corresponding to quartz. A fixed number of particle trajectories were initiated at each release point, 0.5 m above the bottom, every hour for 1 year and then the simulation continued for another year. The AS3D data sets encompassed 1 year and were hence run through twice, updating the velocities, temperatures and densities characterizing the water mass every hour. Tabulated temperature-dependent values of the dynamic viscosity were used in the Stokes formula. The variations in salinity were ignored when this settling velocity was calculated, but the density effects of the stratification were included within the AS3D model framework. The sediment set-up of the TRACMASS model has earlier been briefly described in Kling and Döös (2007).

## Results

**Fig. 3:**
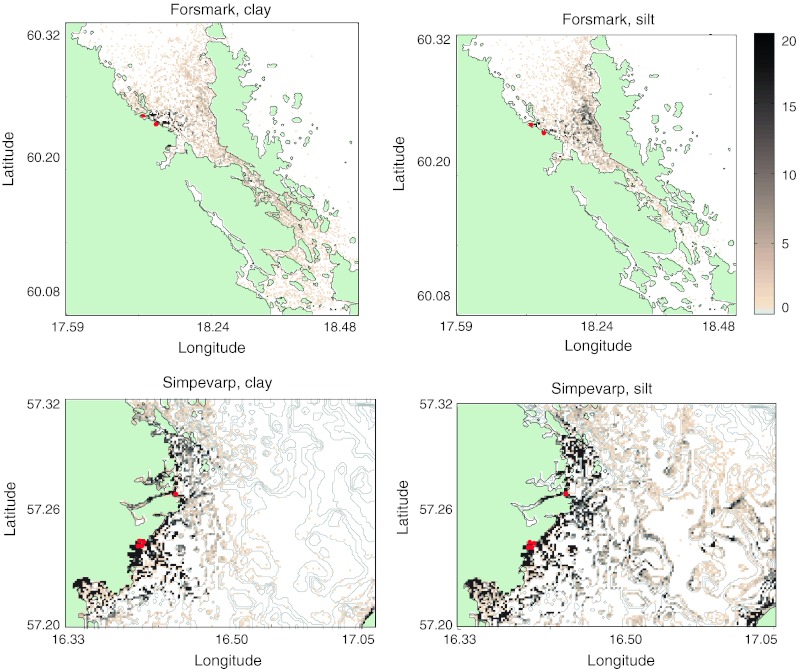
Settled particles after 2 years simulation in Forsmark (*top*) and Simpevarp (*bottom*) for clay (*left*) and silt (*right*). *Red dots* Starting points for the particle trajectories. The color scale is cut off at 20 particles for visibility reasons; in some grid cells the number of particles is much higher

After the 2-year model integration all particles had either settled or left the domains. In the open Simpevarp region most of the particles were transported away, whereas in Forsmark the absolute majority stayed in the domain. In both regions a large proportion of the particles deposited in the release-point grid cells (Table 1). The patterns of the particles that settled within the domain are shown in Fig. 3.

**Table 1:** Left two columns, the percentage of all modeled particles that remained in the absolute vicinity of the release points and that exited the domain. In the right two columns, the average residence time (AvR) until exiting and until first settling event (see also Fig. 4a, b)

	Remain in release point grid cells (%)	Exit domain (%)	AvR (days)	AvR(sed) (days)
Clay Forsmark	60	6	59	15
Silt Forsmark	97	0.1	130	1.0
Clay Simpevarp	4	82	11	19
Silt Simpevarp	30	62	99	3.8

**Fig. 4:**
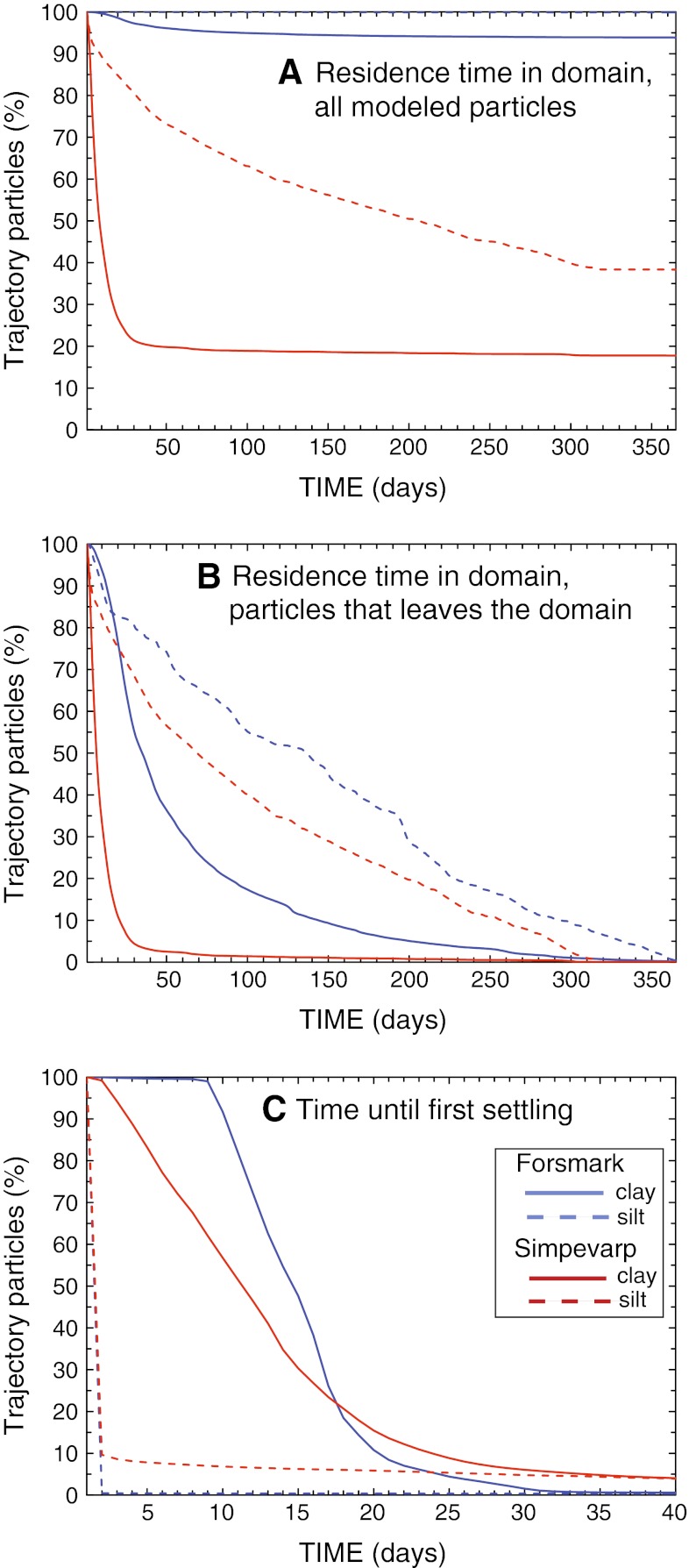
Residence time of the particles, calculated from averages over all 24 × 365 clusters of released particles (every hour for a year)

The residence time in the domain was found by averaging over the entering and exiting times for all particles. The individual times ranged from a day up to more than a year. Figure 4 shows (in percentages) the residence times of particles until exiting or the first settling event and the average values are listed in Table 1. The geographical differences between the areas are visible in the diagrams; Fig. 4b shows how both silt and clay moved slower through the more shielded Forsmark domain, whereas the clay in Simpevarp left during the first month of simulation. The clay in Forsmark remained in suspension for almost 2 weeks before any settling took place (Fig. 4c), while the silt settled during the first day. A small fraction of the silt in Simpevarp stayed in suspension for more than a month and the Simpevarp clay that exited did so within the first 30 days of the simulation (Fig. 4a; Table 1).

**Fig. 5:**
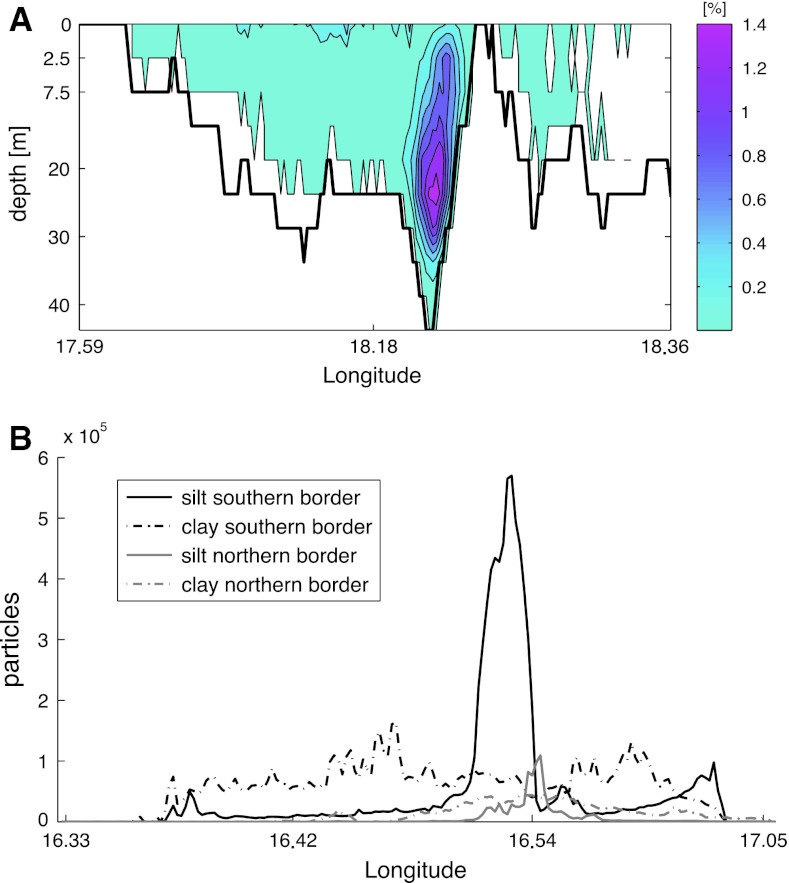
**a** The exit patterns of clay particles in Forsmark; cross-section of the northern border of the domain with the bathymetry as a *black line*. *Color scale* The percentage of all particles that have left, per square meter of border transect, during 2 years of simulation. **b** The amount of clay and silt particles that left the Simpevarp domain through the southern and northern boarders. The eastern border is omitted as the number of particles that exited there was an order of magnitude smaller

The individual-particle tracking permits an analysis of where along the boundaries of the domain the particles exited. This transport was not uniformly distributed; there were a few zones where the bulk of the particles left. Almost 65 % of the Forsmark clay left at a depth of around 20 m, slightly west of the small island Örskär on the northern boundary (Fig. 5a). Almost no silt particles in Forsmark left the domain, but those that did so took the same route as the clay. Despite the openness of the Simpevarp area, the silt particles tended to leave the domain at the middle of the southern border (Fig. 5b) in the uppermost 2.5 m (not shown), whereas the exiting clay was distributed over the entire open boundary.

The distribution of the modeled clay particles in Forsmark was validated against bottom-type data from a survey made by SKB (Elhammar and Sandkvist 2005). The survey data were re-gridded for consistency with the model grid, and a comparison was made with the positions of the modeled clay particles that had settled within the domain. To test the degree of independence between the clay cells in the validation data set and the clay cells in the model data (“clay cell” in the latter case defined as a model grid cell containing at least one clay particle) a standard statistics Pearson’s *χ* ^2^ test was performed. It confirmed dependence (*χ* ^2^ = 24.5, *p* < 0.001) between the model and the validation data. As the modeled particles cluster in some areas of the domain and is absent in others an additional test was performed to test whether the confirmed dependence was on cell level or on area level. This was done by examining pairs of adjacent grid cells, with one cell in the pair being a clay cell in the validation data set and the other a non-clay cell. Using the maximum number of non-overlapping cell pairs it could be shown that the dependence between model particles and validation data clay cells was not at the cell level, but rather at the area level, i.e. the model manages to cluster model particles to validation data clay areas.

## Discussion

We have undertaken a comparison between the large-scale particle transport in two areas with different oceanographic and geomorphological conditions; the particle-release points corresponding to locations of potential discharge of radionuclides in ground water from a future repository. Two particle sizes were modeled and the results were found to differ both between the areas and the particle sizes. In Forsmark the vast majority of the particles stayed in the domain, particularly in shielded coastal areas, where the present-day release points coincide with areas with water velocities too small for any extensive off-shore transport. The Forsmark particles that exited the model domain followed the northward current in the eastern part of the embayment; one of the few places in the domain where the currents are strong enough to result in any substantial particle transport. In the open Simpevarp area most of the particles exited the domain, with a preference for southward flow consonant with the main flow direction seen in Fig. 2.

In both domains the clay particles tended to exit the domain or settle close to the release points, whereas the silt particles were transported farther within the domain once having left the release-point grid cells. The longer residence times for silt, but very short times until first settling imply that the silt particles were transported through repeated settling and resuspension events. In Simpevarp the final silt settling places were in the deeper parts of the basin and the distribution patterns followed the contours of the bottom bathymetry, oriented in such a way that the particles were shielded from the currents. The overall longer residence times in Forsmark were expected, based on the residence times for only water where the Forsmark overturning took 3–6 times longer than in Simepvarp (Engqvist and Andrejev 1999; Engqvist 2006; Engqvist et al. 2006; Döös and Engqvist 2007).

In a safety-assessment context, the particle-transport patterns can provide an impression of the proportion of a potential discharge that would be retained in the near-field, in contrast to leaving the area, if a fraction of the radionuclides entering the sea with the groundwater were particle-reactive enough to adsorb to sediment the particles. For a discussion of the reactiveness of the radionuclides in this safety-assessment study, see Erichsen et al. (2013) and Piqué et al. (2013). In Simpevarp, a larger proportion of a potential discharge would be transported out to open water than in Forsmark, and the site-specific differences in residence times and distribution of settled particles will increase over time as land uplift will make the Forsmark area more secluded from the Baltic Sea (Lindborg 2010; Lindborg et al. 2013).

Clay and silt are rarely transported through the water mass as single particles unless the suspended-sediment concentration is very low, and the particles are generally far from spherical. Our choice of settling velocity parameterization assumes the particles to be smaller (lighter) than they may be, as we neglect flocculation, but due to the larger drag of aggregated and irregular particles our settling velocities are still within the range of in situ measurements. Our criterion for resuspension, taken from Postma (1967), states that a velocity of at least 10 cm s^−1^ is needed for cohesive material. Other studies suggested lower values; e.g., Lam and Jaquet (1976) found a critical velocity of 2–3 cm s^−1^ in freshwater. If the model-generated bottom shear stress had been an output variable in the AS3D circulation model, this quantity would have been a preferable way to handle the deposition and resuspension criteria, as it would permit the use of more modern empirical formulae and in situ measurements.

Wind-induced short surface waves are not included in the AS3D circulation model calculations except as a part of the surface current, and thus the additional orbital velocities associated with these waves are missing. Coupling the AS3D model with a functional wind-wave model would solve this problem, but assuming that highly resolved wind data to force the model were available, this would lead to a pronounced increase in computational demands. Instead calculating the mean fetch and duration of the wind, and employing tabular values of wave properties, could be fruitful in a larger basin such as the entire Baltic Sea, but the mean fetch in Öregrundgrepen is very small in almost every direction. It is mainly in Simpevarp, where the northern and northeastern fetches are large, that the lack of influence by wind-induced short surface waves could make any significant difference. However, as surface-wave effects decrease rapidly with increasing depth, and large parts of the Simpevarp domain are comparatively deep, it would mainly influence the resuspension in the coast-near area. Furthermore, the predominant wind directions in both areas are from the south or southwest (Jönsson et al. 2005; Danielsson et al. 2007), resulting in comparatively small fetches. The pronounced retention of particles near the starting points in Forsmark would probably persist (even with a proper wave model) due to the morphology of the bay where most of the starting points are located. A sub-grid turbulence model would possibly increase the redistribution of particles in areas of low mixing. Such a parameterization is, however, very sensitive to tuning, and might not give any additional accuracy if not correctly tuned. We had no approximations of what would have been appropriate for these areas and at these spatial and temporal resolutions, which militated against using this parameterization. On the whole, there might be too little resuspension in the model. With more resuspension we would have seen a larger transport of sediment away from the start cells into bays and areas along the coastline with weak water motion, as well as into the open part of the domain. The visual patterns (Fig. 3) would probably look approximately the same, although with higher particle concentrations. In this study the cooling water outlets from the power plants are not included. In Forsmark this outflow is located not far from the particle-release points, and might have enhanced the off-shore particle transport had it been included. The circulation model data from the AS3D model was generated for the purpose of describing the flow fields in the two regions independent of the nuclear power plants presently situated there. The results do, however, only represent the effects of the present-day circulation and any detailed prognosis of the future particle patterns cannot be done based solely on this study as the land rise will strongly modify the bathymetry on a timescale of a few thousand years.

## Conclusion

The differences between the areas, one open to the sea and one semi-enclosed, were reflected both in the residence times and in the settling patterns of the particles. In the more confined Forsmark area the clay particles resided on average six times longer than in the open Simpevarp area, 59 days compared with 11. In both domains the silt particles resided much longer before exiting, 138 and 99 days for Forsmark and Simpevarp, respectively. As expected, the fractions of particles exiting the domain were much higher in Simpevarp. The clay that remained in the domains tended to stay close to the release points. Silt particles that did not exit were transported farther within the domains through successive settling and resuspension, particularly in Simpevarp where considerable of along-coast transport and topographically linked settling took place. In Forsmark the vast majority of the sediment particles stayed in the domain, particularly in small inlets with low water velocities. Here only about 6 % of the clay and less than 1 % of the silt exited, whereas in Simpevarp more than 80 % of the clay and 60 % of the silt left the domain. In a safety-assessment perspective the much shorter residence times in Simpevarp are of interest and, also, that a larger proportion of a potential discharge would be transported out to open water there than in Forsmark.

The model did a satisfactory job of reproducing the observed sediment pattern in Forsmark when validated against marine geology survey data. The results of the study reflect the morphological differences between the two domains, and showed a number of distribution and transport patterns that would not been revealed by a diffusive particle model. The differences in particle transport between the domains will increase over time with the land rise that acts faster in Forsmark because of its location closer to the center of the region of greatest isostatic depression during the last glaciation.
